# Association between intraoperative steroid and postoperative mortality in patients undergoing craniotomy for brain tumor

**DOI:** 10.3389/fneur.2023.1153392

**Published:** 2023-06-29

**Authors:** Jialing He, Shuanghong He, Yu Zhang, Yixin Tian, Pengfei Hao, Tiangui Li, Yangchun Xiao, Liyuan Peng, Yuning Feng, Xin Cheng, Haidong Deng, Peng Wang, Weelic Chong, Yang Hai, Lvlin Chen, Chao You, Lu Jia, Dengkui Chen, Fang Fang

**Affiliations:** ^1^Department of Neurosurgery, West China Hospital, Sichuan University, Chengdu, Sichuan, China; ^2^Department of Neurosurgery, The Second Affiliated Hospital of Guangzhou Medical University, Guangzhou, Guangdong, China; ^3^Health Management Center, West China Hospital, Sichuan University, Chengdu, Sichuan, China; ^4^Evidence-Based Medicine Center, Affiliated Hospital of Chengdu University, Chengdu, Sichuan, China; ^5^Department of Neurosurgery, Shanxi Provincial People’s Hospital, Taiyuan, Shanxi, China; ^6^Department of Neurosurgery, Longquan Hospital, Chengdu, Sichuan, China; ^7^Affiliated Hospital of Chengdu University, Chengdu, Sichuan, China; ^8^Department of Medical Oncology, Thomas Jefferson University, Philadelphia, PA, United States; ^9^Sidney Kimmel Medical College, Thomas Jefferson University, Philadelphia, PA, United States; ^10^Department of Neurosurgery, Sichuan Friendship Hospital, Chengdu, Sichuan, China

**Keywords:** brain tumor, steroid administration, mortality, craniotomy, intraoperative period

## Abstract

**Background:**

Despite the widespread use of intraoperative steroids in various neurological surgeries to reduce cerebral edema and other adverse symptoms, there is sparse evidence in the literature for the optimal and safe usage of intraoperative steroid administration in patients undergoing craniotomy for brain tumors. We aimed to investigate the effects of intraoperative steroid administration on postoperative 30-day mortality in patients undergoing craniotomy for brain tumors.

**Methods:**

Adult patients who underwent craniotomy for brain tumors between January 2011 to January 2020 were included at West China Hospital, Sichuan University in this retrospective cohort study. Stratified analysis based on the type of brain tumor was conducted to explore the potential interaction.

**Results:**

This study included 8,663 patients undergoing craniotomy for brain tumors. In patients with benign brain tumors, intraoperative administration of steroids was associated with a higher risk of postoperative 30-day mortality (adjusted OR 1.98, 95% CI 1.09–3.57). However, in patients with malignant brain tumors, no significant association was found between intraoperative steroid administration and postoperative 30-day mortality (adjusted OR 0.86, 95% CI 0.55–1.35). Additionally, administration of intraoperative steroids was not associated with acute kidney injury (adjusted OR 1.11, 95% CI 0.71–1.73), pneumonia (adjusted OR 0.89, 95% CI 0.74–1.07), surgical site infection (adjusted OR 0.78, 95% CI 0.50–1.22) within 30 days, and stress hyperglycemia (adjusted OR 1.05, 95% CI 0.81–1.38) within 24 h after craniotomy for brain tumor.

**Conclusion:**

In patients undergoing craniotomy for benign brain tumors, intraoperative steroids were associated with 30-day mortality, but this association was not significant in patients with malignant brain tumors.

## Introduction

Multiple guidelines recommend the use of steroid administration to provide temporary relief of symptoms related to cerebral edema secondary to the mass effect of brain tumors (1–3). Although the benefit of steroids for this indication is well-established, it also has some serious side effects, including immunosuppression, hyperglycemia, insulin resistance, and poor wound healing (4, 5).

Despite their widespread use during the intraoperative period of neurological surgeries, (6, 7) there is sparse evidence in the literature for the optimal and safe usage of intraoperative steroid administration in patients undergoing craniotomy. Given these serious side effects, there is a requirement to balance the benefit of steroid administration in relieving cerebral edema with the postoperative mortality resulting from steroid-related complications. Due to the immunosuppression of steroids, (8) the association between intraoperative steroid administration and postoperative mortality may be modified by different types of brain tumors.

To fulfill this research gap, we aimed to assess the effects of intraoperative steroid administration on the short-term mortality of patients undergoing craniotomy. Due to its immunosuppression, our study further explored the differences between intraoperative steroid administration and different types of brain tumors.

## Methods

### Study design and data source

We performed an observational and retrospective cohort study approved by the committee of West China Hospital with a waiver of informed consent. A total of 8,663 consecutive electronic health records of patients undergoing craniotomy for brain tumors from West China Hospital, Sichuan University, were collected between January 2011 to January 2020.

### Patient selection

Patients undergoing craniotomy for brain tumors were identified based on the International Classification of Disease, 10th Revision (ICD-10), and procedural codes. We excluded the following patients: (1) age < 18 years old; (2) undergoing craniotomy for pituitary tumor; (3) repeated craniotomy or bur hole procedures; (4) unavailable data of intraoperative electronic anesthesia record; (5) undergoing urgent or emergent surgery; (6) patients whose personal identification number was not found in the electronic health record.

### Baseline characteristics

Preoperative baseline characteristics of patients, including age, gender, cigarette-smoking status (nonsmoker, current smoker, former), alcohol consumption, past medical history (hypertension, diabetes, chronic liver disease, coronary heart disease), and laboratory values (white blood cell counts, blood glucose, total cholesterol, lymphocyte counts, albumin, and hematocrit) were collected. Additional pertinent data on systolic blood pressure, the Charlson Comorbidity Index (CCI), type of brain tumor, histopathology of brain tumor, brain tumor location, brain tumor size, preoperative Karnofsky Performance Status (KPS) score, preoperative steroid use, intraoperative surgery time, intraoperative blood loss, grade of resection, and postoperative mannitol were also recorded.

### Covariate assessment

The primary exposure variable was defined as the intraoperative single-dose administration of various steroids, as indicated in the electronic anesthesia record. Intraoperative steroid administration was compared to no steroid administration. After steroid doses were uniformly converted to dexamethasone, the administered steroid doses were dichotomized into low and high based on the median steroid dose (15 mg dexamethasone) as the cutoff in our study. The dosage of intraoperative steroids was determined based on the severity of the symptoms according to the guideline (1). Additionally, in our hospital, patients who use intraoperative steroids routinely continue to use steroids after craniotomy, and then gradually reduce the dose. The diagnosis of the tumor was identified by the diagnostic text or International Classification of Diseases, 10th revision (ICD-10) codes: Benign (D32–D35, D42–44) or malignant (C70, C71, C75.1–C75.3).

### Outcomes

The primary outcome was 30 days mortality after craniotomy. The secondary outcomes were major postoperative complications including acute kidney injury, pneumonia, surgical site infection within 30 days, and hyperglycemia (≥ 10.0 mmol/L) within 24 h after craniotomy.

Mortality data were acquired from the Household Registration Administration System, also called The Chinese Hukou System. This system is based on governmental statistics management, which uses special personal identification numbers as retrieval keys to find pertinent personal data. When a citizen dies in China, the law mandates the head of household, relatives, dependents, or neighbors to legally notify the death data to the household registration authorities within 1 month (9, 10). In 2021, this system has been updated with data from the Seventh National Census. The National Bureau of Statistics reported that the missing registration rate of the seventh national census was 0.05% (11). Thus, recorded death data in this system were accurate and complete.

### Statistical analysis

Continuous variables were provided as mean ± standard deviation (SD) and categorical variables were summarized using frequencies (%) to describe the distributions of the study population’s demographic and surgical-related data. As appropriate, the comparison of differences between groups was analyzed using the one-way analysis of variance (ANOVA) or the Chi-square test. Multiple imputations were conducted to replace missing values.

Outcomes were assessed by logistic regression models with an adjustment for age, gender, cigarette-smoking status, alcohol consumption, hypertension, diabetes, chronic liver disease, coronary heart disease, CCI, type of brain tumor, brain tumor location, brain tumor size, systolic blood pressure, preoperative KPS score, preoperative steroid use, intraoperative surgery time, intraoperative blood loss, grade of resection, and postoperative mannitol. Variables with *p* < 0.10 were implemented into a backward stepwise multivariable logistic regression model to explore the association between steroid administration and outcomes. Multicollinearity was evaluated by computing the variance inflation factor (VIF) for all variables, and when VIF was higher than 5, there was significant multicollinearity that needed to be corrected. The propensity scores matching (12, 13) was performed as the sensitivity analysis with a matching ratio (Steroids: No Steroids) of 1:1 and a caliper distance of 0.2.

We performed subgroup analyses by dividing patients into groups of those who received steroids and those who did not to investigate if there were any variations in the association according to different baseline variables.

R statistical software (version 4.2.1; Foundation for Statistical Computing) was used to perform all statistical analyses. Bonferroni *p* < 0.01 were considered statistically significant for the subgroup analyses. In other analyses, statistical significance was set as a 2-tailed value of *p* less than 0.05.

## Results

### Baseline characteristics

Baseline characteristics for the 8,663 patients who met the inclusion criteria (Supplementary Figure S1) of this study are displayed in Table 1. A total of 6,307 (72.8%) patients received intraoperative steroids, and patients presenting with malignant brain tumors were more prone to receive steroids (steroid use accounted for 67.6% of all patients with benign brain tumors and 80.7% for all patients with malignant brain tumors).

**Table 1:** Baseline characteristics of study population.

Characteristics	No Steroids (*n* = 2,356)	Steroids^*^ (*n* = 6,307)	*P*
Age (>65), *n* (%)	325 (13.8)	773 (12.3)	0.06
Female, *n* (%)	1,446 (61.4)	3,514 (55.7)	<0.001
Cigarette-smoking status, *n* (%)			<0.001
Nonsmoker	1937 (82.2)	4,792 (76.0)	
Current smoker	230 (9.8)	843 (13.4)	
Former	189 (8.0)	672 (10.7)	
Alcohol consumption, *n* (%)	358 (15.2)	1,223 (19.4)	<0.001
Medical history, *n* (%)			
Hypertension	377 (16.0)	904 (14.3)	0.06
Diabetes	165 (7.0)	371 (5.9)	0.06
Chronic liver disease	112 (4.8)	288 (4.6)	0.76
Coronary heart disease	29 (1.2)	52 (0.8)	0.10
CCI score, *n* (%)			<0.001
0	1,472 (62.5)	3,109 (49.3)	
≥ 1	884 (37.5)	3,198 (50.7)	
Type of brain tumor, *n* (%)			<0.001
Benign	1,696 (72.0)	3,539 (56.1)	
Malignant	660 (28.0)	2,768 (43.9)	
Histopathology, *n* (%)			<0.001
Schwannoma	235 (10.0)	431 (6.8)	
Meningioma	1,016 (43.1)	2,174 (34.5)	
Craniopharyngioma	22 (0.9)	45 (0.7)	
Glioma WHO grades I–II	446 (18.9)	1,119 (17.7)	
Glioma WHO grades III–IV	427 (18.1)	1927 (30.6)	
Metastasis	127 (5.4)	437 (6.9)	
Other	83 (3.5)	174 (2.8)	
Brain tumor location, *n* (%)			<0.001
basal ganglia	1 (0.0)	8 (0.1)	
Brainstem	37 (1.6)	176 (2.8)	
Cerebellum	150 (6.4)	326 (5.2)	
Convexity	1,257 (53.4)	3,881 (61.5)	
Skull base	815 (34.6)	1,560 (24.7)	
Ventricle	78 (3.3)	335 (5.3)	
Other	18 (0.8)	21 (0.3)	
Brain tumor size, mean (SD), cm^3^	22.6 (35.8)	27.5 (39.7)	<0.001
Preoperative SBP, mmHg, mean (SD)	125.8 (16.3)	124.6 (16.2)	0.01
Preoperative laboratory, mean (SD)			
WBC counts, 10^9^/L	11.0 (4.5)	12.2 (5.0)	<0.001
Blood glucose, mmol/L	6.4 (2.0)	6.6 (2.2)	0.01
Lymphocyte counts, 10^9^/L	1.2 (0.6)	1.3 (0.8)	0.04
Albumin, g/L	37.3 (4.9)	37.7 (4.9)	0.01
Hematocrit	0.4 (0.1)	0.4 (0.1)	<0.001
Preoperative KPS score, *n* (%)			<0.001
0–50	229 (9.7)	1,096 (17.4)	
51–80	1,306 (55.4)	2,692 (42.7)	
81–100	821 (34.8)	2,518 (39.9)	
Preoperative steroid use, *n* (%)	1884 (80.0)	5,915 (93.8)	<0.001
Intraoperative, mean (SD)			
Surgery time, hours	3.89 (1.95)	4.01 (1.83)	0.01
Blood loss, ml	327.7 (475.5)	338.0 (492.0)	0.40
Grade of resection, *n* (%)			
Gross total resection	1748 (74.2)	4,277 (67.8)	<0.001
Subtotal resection	608 (25.8)	2030 (32.2)	
Postoperative mannitol, ml, mean (SD)	387.0 (455.1)	663.0 (444.9)	<0.001

^*^Intraoperative; CCI, Charlson comorbidity index; SBP, Systolic blood pressure; SD, standard deviation; KPS, Karnofsky Performance Status; WBC, White blood cell; WHO, World health organization.

Number of miss value (%): KPS 1 (0.0).

### Primary analysis

**Figure 1:**
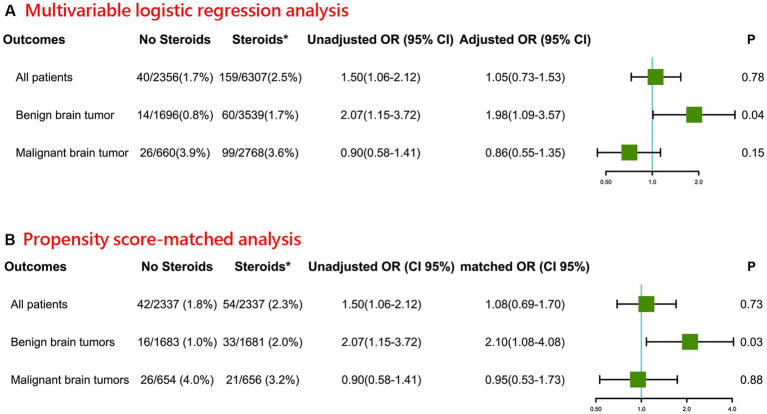
Association between intraoperative steroid administration and postoperative 30-day mortality of patients undergoing craniotomy for brain tumor **(A)** Multivariable logistic regression analysis. **(B)** Propensity score-matched analysis (*Intraoperative; OR: Odds ratio for comparing Steroids group vs. No Steroids group).

As shown in Figure 1, a total of 199 (2.3%) patients died within 30 days of their craniotomy, of whom 159 received intraoperative steroids, and 40 did not. In the multivariable logistic regression analysis, for all patients with benign or malignant brain tumors, the univariate logistic analysis showed that patients who received intraoperative steroids had higher odds of 30-day postoperative mortality (OR 1.50, 95% CI 1.06–2.12). However, after adjusting for CHD, CCI score, type of brain tumor, location of brain tumor, preoperative KPS score, intraoperative surgery time, intraoperative blood loss, and postoperative mannitol, this association was not significant (adjusted OR 1.05, 95% CI 0.73–1.53). The details of the regression model were presented in Supplementary Table S1. No obvious multicollinearity was detected in the regression model of our study (all VIFs <5).

Furthermore, the intraoperative administration of steroids in patients with benign brain tumors was associated with a higher risk of postoperative 30-day mortality (adjusted OR 1.98, 95% CI 1.09–3.57). However, in patients with malignant brain tumors, no significant association was found between intraoperative steroid administration and postoperative 30-day mortality (adjusted OR 0.86, 95% CI 0.55–1.35).

### Sensitivity analyses

For sensitivity analyses, we further applied a propensity score-matched (1:1) analysis to validate our obtained results (Figure 1). Baseline characteristics of propensity score–matched samples were displayed in Supplementary Table S2. Consistent with the primary analysis, the intraoperative administration of steroids in patients with benign brain tumors was associated with higher odds of postoperative 30-day (matched OR 2.10, 95% CI 1.08–4.08). Likewise, in patients with malignant brain tumors, there was no statistical difference between intraoperative steroids administration and postoperative 30-day mortality (matched OR 0.95, 95% CI 0.53–1.73).

### Postoperative complications

**Figure 2:**
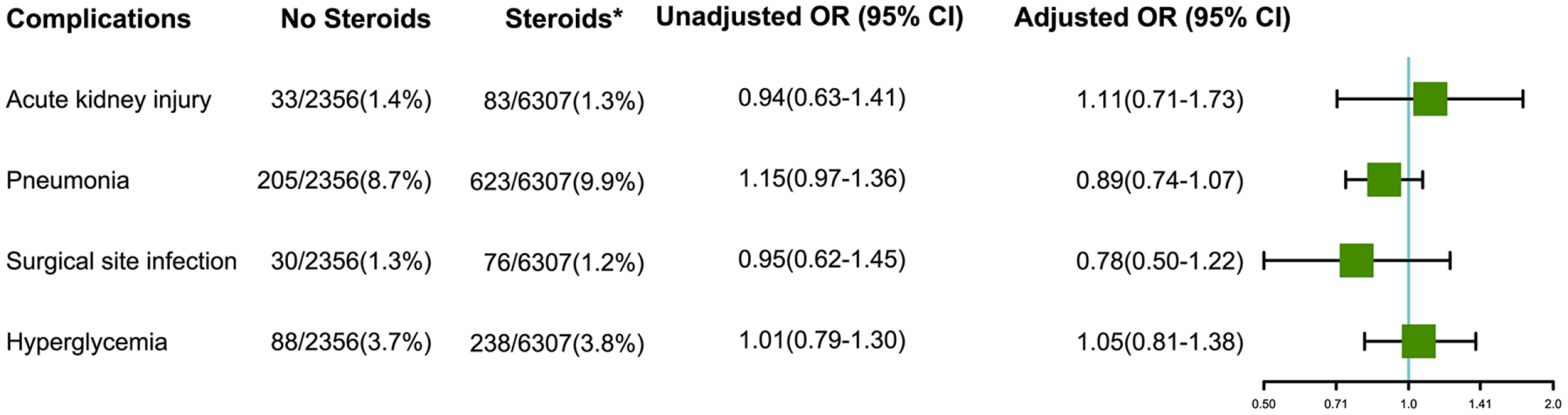
Effects of intraoperative steroids on postoperative complications after craniotomy for brain tumors (*Intraoperative; OR: Odds ratio for comparing Steroids group vs. No Steroids group).

The results of other postoperative complications were shown in Figure 2. Administration of intraoperative steroids was not associated with acute kidney injury (adjusted OR 1.11, 95% CI 0.71–1.73), pneumonia (adjusted OR 0.89, 95% CI 0.74–1.07), surgical site infection (adjusted OR 0.78, 95% CI 0.50–1.22) within 30 days, and stress hyperglycemia (adjusted OR 1.05, 95% CI 0.81–1.38) within 24 h after craniotomy for brain tumor.

### Subgroup analyses and stratified analyses by steroids dose/types

We conducted subgroup analyses and further explored interactions between different variables and intraoperative administration of steroids regarding the outcome of postoperative 30-day mortality in Supplementary Figure S2. There were no significant interactions in this study.

**Figure 3:**
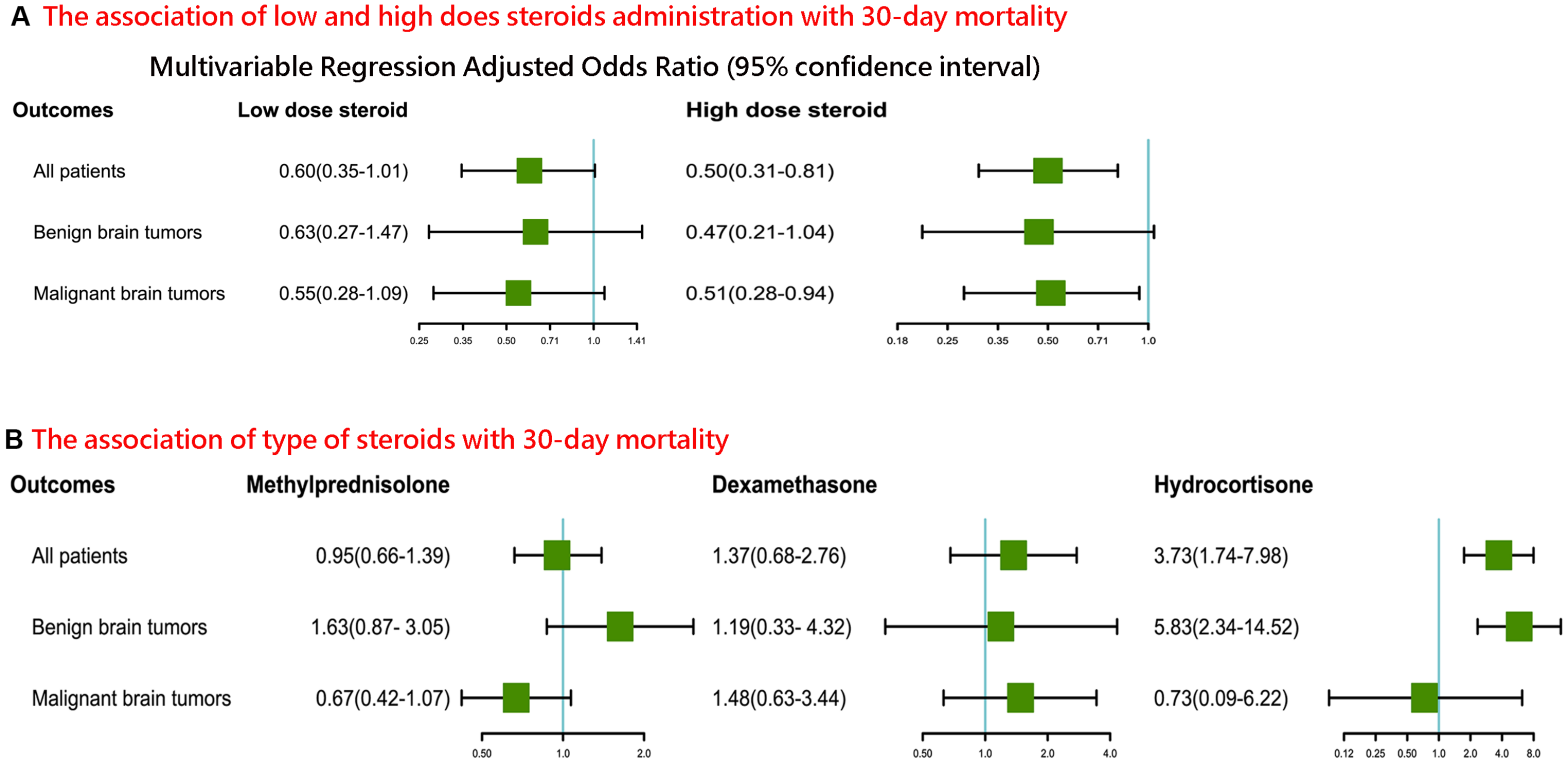
Associations of steroids dose **(A)** and types **(B)** with postoperative 30-day mortality in patients undergoing craniotomy for brain tumor.

After adjustment for confounders, among patients with malignant brain tumors, a higher (≥15 mg dexamethasone) steroid dose was associated with lower 30-day postoperative mortality after craniotomy (adjusted OR 0.51, 95% CI 0.28–0.94, Figure 3), but this association was not found among patients with benign brain tumors (adjusted OR 0.47, 95% CI 0.21–1.04), compared with those who did not receive intraoperative steroids. Additionally, based on the cutoff dose of 10 mg dexamethasone, these associations were still found (Supplementary Table S3).

Compared with patients who did not receive intraoperative steroids, those with benign brain tumors who received hydrocortisone were significantly associated with postoperative 30-day mortality (adjusted OR 5.83, 95% CI 2.34–14.52, Figure 3), but not for patients who received methylprednisolone (adjusted OR 1.63, 95% CI 0.87–3.05) and dexamethasone (adjusted OR 1.19, 95% CI 0.33–4.32). Additionally, compared with patients who received dexamethasone, no significant difference in postoperative 30-day mortality was detected among patients who received methylprednisolone (adjusted OR 0.71, 95% CI 0.37–1.36).

## Discussion

In this study of 8,663 patients undergoing craniotomy for brain tumors, patients with benign brain tumors who received intraoperative steroids were associated with an increased risk of postoperative 30-day mortality, but this association was not found in patients with malignant brain tumors.

Studies investigating the effects of perioperative steroid administration for neurosurgical procedures have shown conflicting results. In a retrospective analysis of 435 patients undergoing resection of primary glioma, it was suggested that preoperative dexamethasone use could potentially have a negative impact on survival (14). However, the findings of this study were limited by its small sample size. In contrast, another retrospective study involving 4,407 patients undergoing craniotomy for malignant brain tumors found that preoperative steroids did not increase the risk of postoperative 30-day mortality (15). However, this study did not assess the effects of steroid administration on postoperative 30-day mortality in patients undergoing craniotomy for benign brain tumors.

To our knowledge, our study is the first to investigate the association between intraoperative steroids and postoperative 30-day mortality in patients undergoing craniotomy for brain tumors. This study benefited from a large sample size, allowing for more precise statistical analyses of 30-day postoperative mortality with a low incidence (2.4%). Furthermore, we conducted propensity score-matched analyses to minimize potential selection bias that may result from the observed covariates. It was important to note that the accuracy of death records in our study was ensured by utilizing household registration data administered by the Chinese government.

Several mechanisms may explain the increased postoperative mortality of patients who received intraoperative steroids undergoing craniotomy for brain tumors. First, most patients have increased serum glucose levels after receiving steroids (16), and hyperglycemia may result in shorter survival in glioblastoma patients (17). Second, steroid use is associated with osteoporosis due to the induction of apoptosis in osteoblasts and osteocytes (18) and a reduction of cytokine-dependent osteoblast differentiation (19). Patients with steroid-induced osteoporosis may have a higher mortality rate (20, 21).

Interestingly, we found that among patients with malignant brain tumors, a higher (≥15 mg dexamethasone) steroid dose was associated with lower 30-day postoperative mortality after craniotomy, but a lower (<15 mg dexamethasone) steroid dose was not. One possible explanation for this finding was that low-dose steroids might not be sufficient to control inflammation and brain swelling. In contrast, high-dose intraoperative steroids (≥15 mg) have been shown to be safe and effective in reducing inflammation and brain swelling, and might even be associated with improved patient outcomes. More studies in the future are needed to explore the potential mechanisms behind these associations.

### Limitations

Our study has several limitations. First, the retrospective design of this study may introduce potential confounders and biases. For example, there may be selection bias in which tumors that require an approach through or close to vital structures are more likely to receive steroids. Additionally, the surgeon’s reasoning when deciding to use intraoperative steroids, adjuvant chemotherapy and/or radiotherapy, and adjuvant treatment side effects could be potential confounders. Moreover, we were unable to obtain data on some important outcomes such as adjuvant treatment success in controlling tumor growth and tumor recurrence. Second, most patients in our study received methylprednisolone instead of dexamethasone which is recommended by the guidelines (1, 22). However, no statistical difference in postoperative 30-day mortality was found between the two types of steroids in our study. Third, different histopathologic types of tumors have different resection scales, which may impact adjuvant treatment type and prognosis. However, we only classify them into gross total resection and subtotal resection uniformly. Fourth, all the patients in this study were from a single institution, which may limit the generalizability of our findings.

## Conclusion

In the patients undergoing craniotomy for brain tumors, patients with benign brain tumors who received intraoperative steroids were associated with an increased risk of postoperative 30-day mortality, but this association was not found in patients with malignant brain tumors. The effects of dosage, types, and duration of intraoperative steroids on patients undergoing craniotomy for brain tumors should be considered when designing future prospective studies.

## Data availability statement

The raw data supporting the conclusions of this article will be made available by the corresponding authors.

## Ethics statement

The studies involving human participants were reviewed and approved by the ethics committee of West China Hospital (No. 2022–705). Written informed consent for participation was not required for this study in accordance with the national legislation and the institutional requirements.

## Author contributions

FF: study concept. JH and SH: acquisition, analysis, or interpretation of data, statistical analysis, and drafting of the manuscript. All authors: design and critical revision of the manuscript for important intellectual content.

## Funding

This study was supported by National Natural Science Foundation of China (82271364) YZ, the innovation team project of Affiliated Hospital of Clinical Medicine College of Chengdu University (CDFYCX202203) YZ, and the project of Sichuan Science and Technology Bureau (22ZDYF0798) FF.

## Conflict of interest

The authors declare that the research was conducted in the absence of any commercial or financial relationships that could be construed as a potential conflict of interest.

## Publisher’s note

All claims expressed in this article are solely those of the authors and do not necessarily represent those of their affiliated organizations, or those of the publisher, the editors and the reviewers. Any product that may be evaluated in this article, or claim that may be made by its manufacturer, is not guaranteed or endorsed by the publisher.
