# Supplementary material for: Association between intraoperative steroid and postoperative mortality in patients undergoing craniotomy for brain tumor
Source: Front Neurol. 2023 Jun 29;14:1153392. doi: 10.3389/fneur.2023.1153392 (PMC10339830; doi:10.3389/fneur.2023.1153392)
Supplement: Supplementary file 2 [file Data_Sheet_1.docx]

Data Sheet 1


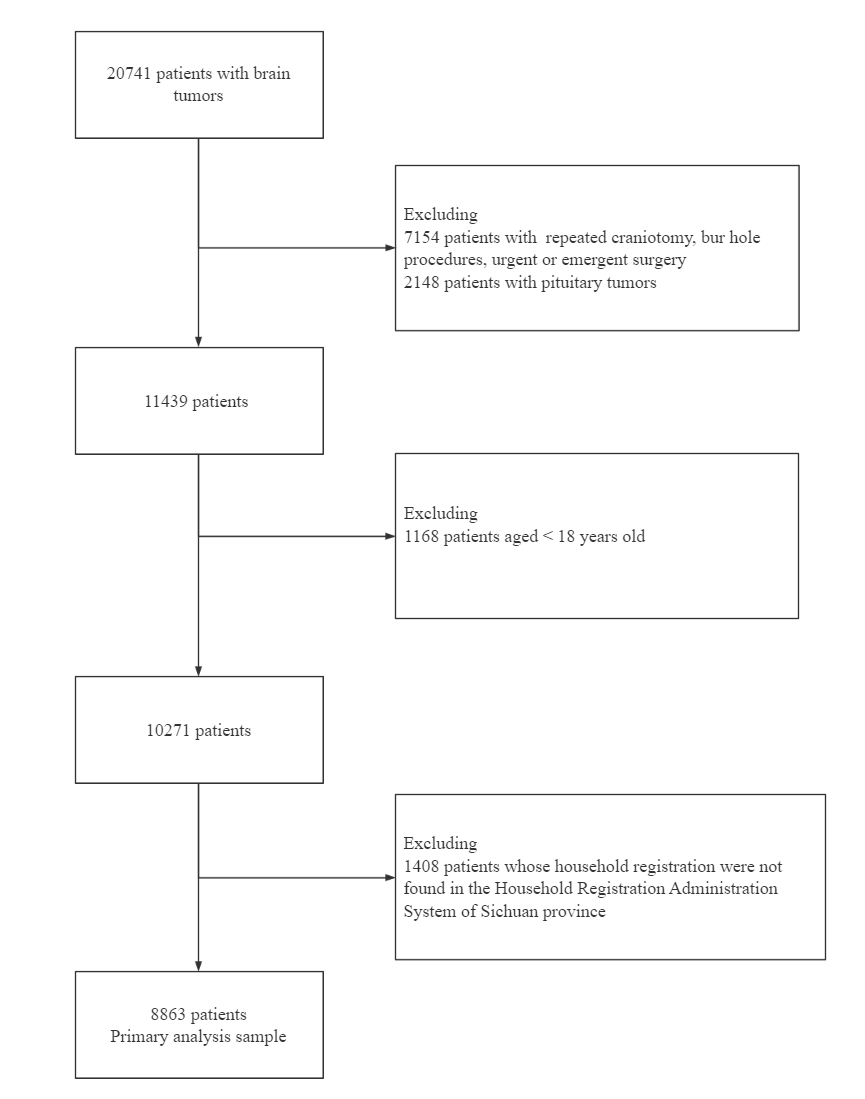


**eFigure 1 Flowchart of Patient Selection**

**
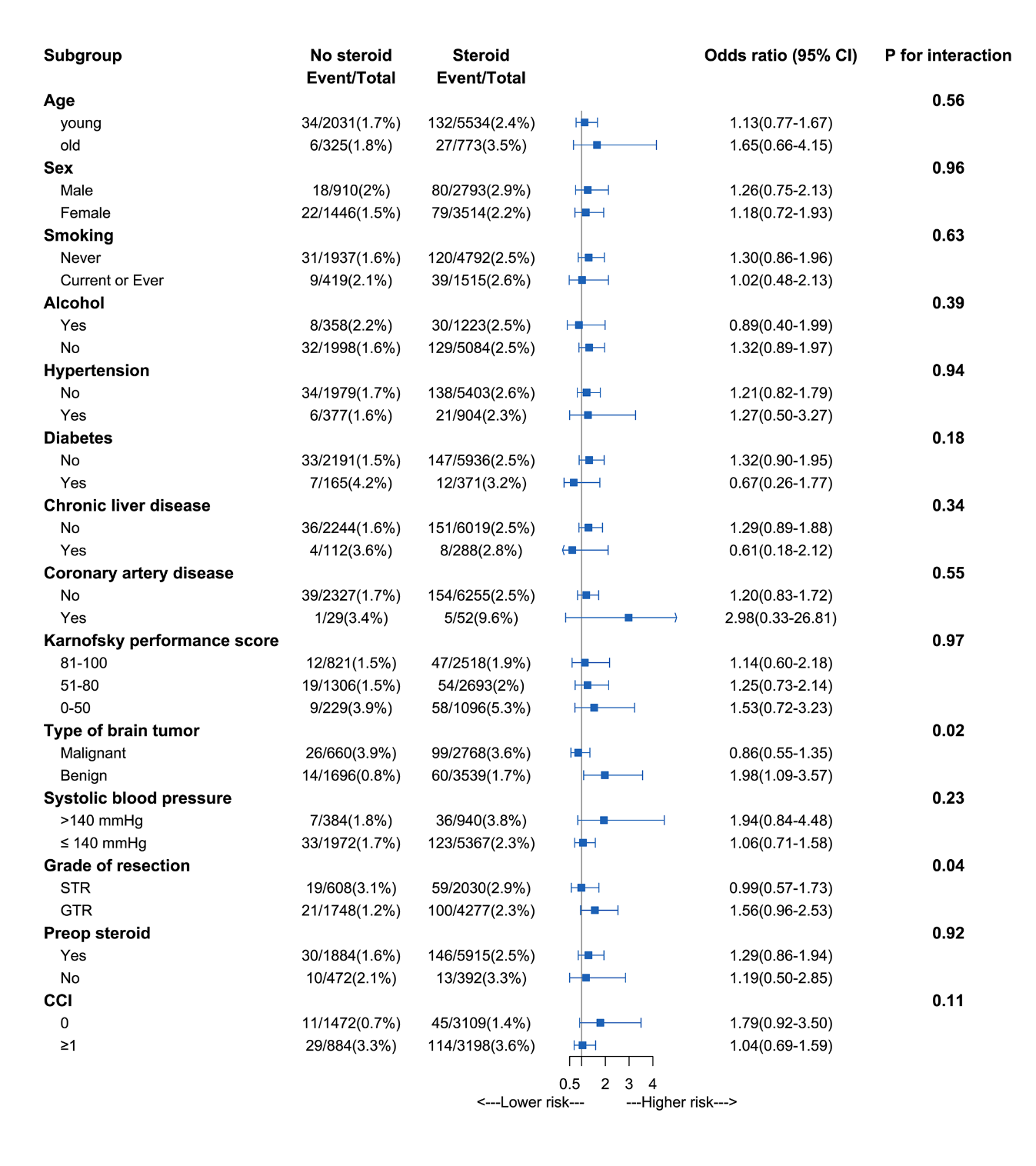
**

**eFigure 2 Subgroup analysis of the association between intraoperative steroids administration and 30-day mortality**

**
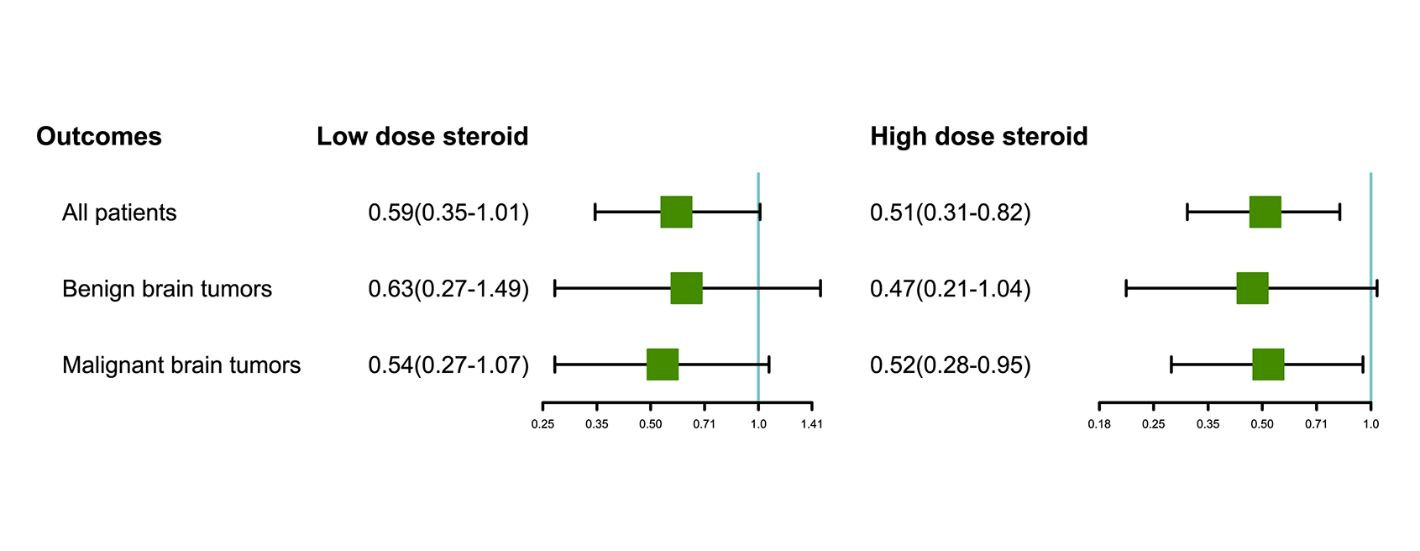
**

**eFigure 3 The association of low (<10mg) and high (≥10mg) dose steroids administration with 30-day mortality**

Multivariable regression adjusted odds ratio (95% confidence interval)

**eTable 1 Regression analysis for 30-day mortality in patients with benign brain tumors**

| **Characteristics** | **Unadjusted** | | **Adjustment** | |
| --- | --- | --- | --- | --- |
|  | OR (95% CI) | P | OR (95% CI) | P |
| **Age (>65 years)** | 1.38(0.95- 2.02) | 0.10 | NA | NA |
| **Female** | 0.76(0.58- 1.01) | 0.06 | NA | NA |
| **Cigarette-smoking status** |  |  |  | NA |
| Nonsmoker | 1 [Reference] |  | 1 [Reference] |  |
| Current smoker | 0.83(0.52- 1.32) | 0.43 | NA | NA |
| Former | 1.46(0.97- 2.21) | 0.07 | NA | NA |
| **Alcohol consumption** | 1.06(0.74- 1.51) | 0.76 | NA | NA |
| **Medical history** |  |  |  |  |
| Hypertension | 0.90(0.60- 1.36) | 0.62 | NA | NA |
| Diabetes | 1.62(1.00- 2.62) | 0.05 | NA | NA |
| Chronic liver disease | 1.34(0.74- 2.42) | 0.34 | NA | NA |
| Coronary heart disease | 3.48(1.50- 8.08) | 0.00 | 2.92(1.20- 7.12) | 0.02 |
| **CCI score** |  |  |  |  |
| 0 | 1 [Reference] |  | 1 [Reference] |  |
| ≥ 1 | 2.93(2.15- 4.01) | <0.001 | 1.98(1.17- 3.34) | 0.01 |
| **Type of brain tumor** |  |  |  |  |
| Benign | 1 [Reference] |  | 1 [Reference] |  |
| Malignant | 2.64(1.97- 3.53) | <0.001 | 1.64(0.98- 2.76) | 0.06 |
| **Brain tumor location** |  |  |  |  |
| basal ganglia | 1 [Reference] |  | 1 [Reference] |  |
| Brainstem | 1.31(0.16-10.87) | 0.80 | 1.76(0.21-15.10) | 0.60 |
| Cerebellum | 0.22(0.03- 1.93) | 0.17 | 0.53(0.06- 4.69) | 0.57 |
| Convexity | 0.14(0.02- 1.11) | 0.06 | 0.29(0.03- 2.43) | 0.25 |
| Skull base | 0.15(0.02- 1.23) | 0.08 | 0.52(0.06- 4.49) | 0.55 |
| Ventricle | 0.49(0.06- 4.11) | 0.51 | 1.10(0.13- 9.51) | 0.93 |
| Other | 0.00(0.00- Inf) | 0.97 | 0.00(0.00- Inf) | 0.97 |
| **Brain tumor size** | 1.00(1.00- 1.00) | 0.77 | 1.00(1.00- 1.00) | 0.95 |
| **Preoperative SBP** | 1.00(0.99- 1.01) | 0.81 | NA | NA |
| **Preoperative KPS score** |  |  |  |  |
| 0-50 | 1 [Reference] |  | 1 [Reference] |  |
| 51-80 | 0.35(0.25- 0.49) | <0.001 | 0.52(0.36- 0.73) | <0.001 |
| 80-100 | 0.34(0.24- 0.48) | <0.001 | 0.35(0.24- 0.52) | <0.001 |
| **Preoperative steroid** | 0.84(0.54- 1.31) | 0.45 | NA | NA |
| **Intraoperative** |  |  |  |  |
| Surgery time, hours | 1.14(1.07- 1.21) | <0.001 | 1.13(1.05- 1.22) | <0.001 |
| Blood loss, ml | 1.00(1.00- 1.00) | <0.001 | 1.00(1.00- 1.00) | <0.001 |
| **Grade of resection** |  |  |  |  |
| Subtotal resection | 1 [Reference] |  | 1 [Reference] |  |
| Gross total resection | 0.67(0.50- 0.90) | 0.01 | NA | NA |
| **Postoperative mannitol** | 1.00(1.00-1.00) | <0.001 | 1.00(1.00-1.00) | 0.01 |
| **Intraoperative steroids use** | 1.50(1.06- 2.12) | 0.02 | 1.05(0.73- 1.53) | 0.78 |

CCI: Charlson comorbidity index; SBP: systolic blood pressure; KPS: Karnofsky Performance Status

**eTable 2 Baseline characteristics of Propensity Score–Matched Samples**

|  | **Before matching** | | | **After matching** | | |
| --- | --- | --- | --- | --- | --- | --- |
| **Demographics** | **No Steroids  (n = 2356)** | **Steroids*  (n = 6307)** | **SMD** | **No Steroids  (n = 2169)** | **Steroids*  (n = 2169)** | **SMD** |
| **Age (>65years), n (%)** | 325 (13.8) | 773 (12.3) | 0.05 | 299 (13.8) | 305 (14.1) | 0.01 |
| **Female, n (%)** | 1446 (61.4) | 3514 (55.7) | 0.12 | 1317 (60.7) | 1330 (61.3) | 0.01 |
| **Cigarette-smoking status, n (%)** |  |  | 0.15 |  |  | 0.05 |
| Nonsmoker | 1937 (82.2) | 4792 (76.0) |  | 1773 (81.7) | 1733 (79.9) |  |
| Current smoker | 230 (9.8) | 843 (13.4) |  | 216 (10.0) | 234 (10.8) |  |
| Former | 189 (8.0) | 672 (10.7) |  | 180 (8.3) | 202 (9.3) |  |
| **Alcohol consumption, n (%)** | 358 (15.2) | 1223 (19.4) | 0.11 | 340 (15.7) | 354 (16.3) | 0.02 |
| **Medical history, n (%)** |  |  |  |  |  |  |
| Hypertension | 377 (16.0) | 904 (14.3) | 0.05 | 348 (16.0) | 358 (16.5) | 0.01 |
| Diabetes | 165 (7.0) | 371 (5.9) | 0.05 | 153 (7.1) | 146 (6.7) | 0.01 |
| Chronic liver disease | 112 (4.8) | 288 (4.6) | 0.01 | 103 (4.7) | 98 (4.5) | 0.01 |
| Coronary heart disease | 29 (1.2) | 52 (0.8) | 0.04 | 26 (1.2) | 32 (1.5) | 0.02 |
| **CCI score** ≥ 1**, n (%)** | 884 (37.5) | 3198 (50.7) | 0.27 | 830 (38.3) | 798 (36.8) | 0.03 |
| **Type of brain tumor, n (%)** |  |  | 0.34 |  |  | 0.01 |
| Benign | 1696 (72.0) | 3539 (56.1) |  | 1539 (71.0) | 1567 (72.2) | 0.03 |
| Malignant | 660 (28.0) | 2768 (43.9) |  | 630 (29.0) | 602 (27.8) |  |
| **Brain tumor location, n (%)** |  |  | 0.26 |  |  | 0.08 |
| basal ganglia | 1 (0.0) | 8 (0.1) |  | 1 (0.0) | 2 (0.1) |  |
| Brainstem | 37 (1.6) | 176 (2.8) |  | 35 (1.6) | 36 (1.7) |  |
| Cerebellum | 150 (6.4) | 326 (5.2) |  | 136 (6.3) | 140 (6.5) |  |
| Convexity | 1257 (53.4) | 3881 (61.5) |  | 1170 (53.9) | 1097 (50.6) |  |
| Skull base | 815 (34.6) | 1560 (24.7) |  | 742 (34.2) | 806 (37.2) |  |
| Ventricle | 78 (3.3) | 335 (5.3) |  | 76 (3.5) | 73 (3.4) |  |
| Other | 18 (0.8) | 21 (0.3) |  | 9 (0.4) | 15 (0.7) |  |
| **Brain tumor size, mean (SD), cm³** | 22.6 (35.8) | 27.5 (39.7) | 0.11 | 23.3 (36.4) | 22.6 (31.1) | 0.02 |
| **Preop SBP, mmHg, mean (SD)** | 125.8 (16.3) | 124.6 (16.2) | 0.07 | 125.8 (16.4) | 125.7 (16.3) | 0.01 |
| **Preoperative KPS score, n (%)** |  |  | 0.29 |  |  | 0.03 |
| 0-50 | 229 (9.7) | 1096 (17.4) |  | 223 (10.3) | 229 (10.6) |  |
| 51-80 | 1306 (55.4) | 2693 (42.7) |  | 1173 (54.1) | 1146 (52.8) |  |
| 80-100 | 821 (34.8) | 2518 (39.9) |  | 773 (35.6) | 794 (36.6) |  |
| **Preoperative steroid use, n (%)** | 1884 (80.0) | 5915 (93.8) | 0.42 | 1880 (86.7) | 1855 (85.5) | 0.03 |
| **Intraoperative, mean (SD)** |  |  |  |  |  |  |
| Surgery time, hours | 3.9 (2.0) | 4.0 (1.8) | 0.06 | 3.9 (2.0) | 4.0 (2.0) | 0.03 |
| Blood loss, ml | 327.7 (475.5) | 338.0 (492.0) | 0.03 | 321.3 (459.8) | 335.4 (508.1) | 0.03 |
| **Grade of resection** |  |  | 0.14 |  |  | 0.05 |
| Gross total resection | 1748 (74.2) | 4277 (67.8) |  | 1600 (73.8) | 1649 (76.0) |  |
| Subtotal resection | 608 (25.8) | 2030 (32.2) |  | 569 (26.2) | 520 (24.0) |  |
| **Postop mannitol, ml, mean (SD)** | 387.0 (455.1) | 663.0 (444.9) | 0.61 | 415.0 (457.0) | 443.5 (405.9) | 0.07 |

*Intraoperative

^#^A value of the standard median difference < 0.1 indicates covariate balance
CCI: Charlson comorbidity index; SBP: systolic blood pressure; KPS: Karnofsky Performance Status
